# Assessing the Quality, Privacy, and Security of Breast Cancer Apps for Arabic Speakers: Systematic Search and Review of Smartphone Apps

**DOI:** 10.2196/48428

**Published:** 2024-01-16

**Authors:** Dari Alhuwail, Aisha Alhouti, Latifah Alsarhan

**Affiliations:** 1 Information Science Department College of Life Sciences Kuwait University Sabah AlSalem University City Kuwait; 2 Health Informatics Unit Dasman Diabetes Institute Dasman Kuwait

**Keywords:** apps, Arabic, awareness, breast cancer, consumer health informatics, education, mHealth, mobile health, privacy, quality, security, smartphone, women

## Abstract

**Background:**

Breast cancer is a widespread disease, and its incidence is rapidly increasing in the Middle East and North Africa region. With the increasing availability of smartphone apps for various health purposes, breast cancer apps have emerged as tools for raising awareness, providing support, and empowering women affected by this disease. These apps offer many features, including information on breast cancer risk factors, self-examination guides, appointment reminders, and community support groups or hotlines. Using apps raises the risk of privacy and security issues, and we hope that examining these features of the apps will contribute to the understanding of how technology can be used to improve these apps and provide insights for future development and improvement of breast cancer apps.

**Objective:**

This study aims to critically review the quality, privacy, and security of breast cancer apps available to Arabic speakers.

**Methods:**

Similar to several recent studies, we used a systematic search for apps available in Google Play and Apple App stores using both the web interface and the built-in native stores installed on smartphones. The search was conducted in mid-December 2022 in Arabic using the following keywords: سرطان الثدي – فحص سرطان الثدي – علاج سرطان الثدي – مرض سرطان الثدي – أعراض سرطان الثدي – فحص الثدي (breast cancer, breast cancer treatment, breast cancer disease, breast cancer symptoms, breast cancer screening, and breast test). These preidentified search terms are based on earlier work concerning the top searched breast cancer topics by Arabic speakers through Google’s search engine. We excluded apps that did not have an Arabic interface, were developed for non-Arabic speakers, were paid, needed a subscription, or were directed toward health care workers. The Mobile App Rating Scale was used to evaluate the quality of the apps concerning their engagement, functionality, aesthetics, and information. A risk score was calculated for the apps to determine their security risk factors.

**Results:**

Only 9 apps were included, with most (6/9, 67%) being supported by advertisements and categorized as informational. Overall, the apps had low numbers of downloads (>10 to >1000). The majority of the included apps (8/9, 89%) requested dangerous access permissions, including access to storage, media files, and the camera. The average security score of the included apps was 3.22, while only 2 apps provided information about data security and privacy. The included apps achieved an overall average quality score of 3.27, with individual dimension scores of 4.75 for functionality, 3.04 for information, 3.00 for aesthetics, and 2.32 for engagement.

**Conclusions:**

The limited availability of breast cancer apps available to Arabic speakers should be a call to action and prompt health care organizations and developers to join forces and collaboratively develop information-rich, usable, functional, engaging, and secure apps.

## Introduction

Female breast cancer is among the most commonly diagnosed cancers worldwide, with a rate of approximately 2.3 million new cases [1]. Over the past decade, breast cancer incidence has been on the rise in the Middle East and North Africa (MENA) region [2]. Breast cancer is the most frequently diagnosed cancer (17.7%-19% of all types of cancer) in the region [3], and it accounts for 30% of all cancer cases [4]. The lack of cancer education and barriers to cancer screening are seen as major health problems [5]. Education is one of the most effective tools in the fight against female breast cancer; it can have positive effects on women’s practices, attitudes, and knowledge of the disease [6]. However, recent studies suggest a lack of resources and poor awareness of breast cancer in women in the MENA region [7]. While the digital delivery of health education content has been on the rise through different digital media (eg, websites and social media), the quality of Arabic content for female breast cancer remains poor [8-10].

Smartphone proliferation, ubiquity, and affordability, as well as the increasing availability of mobile apps, may be the long-awaited for “digiceuticals” or digital therapeutics [11-13]. Today, the number of health apps in smartphone app stores exceeds 325,000 [14] and will continue to rise, with estimates of more than 200 apps being added daily to app stores [15], covering a wide spectrum of health purposes, such as well-being, education, and disease management, including chronic conditions [16]. Several apps are available that can help individuals with breast cancer manage their condition. These apps have been used for the purposes of education [17-19], care management [20,21], prevention [22-24], and well-being [25,26]. These apps can be a valuable resource for individuals with breast cancer, helping them stay informed and connected to their care team and manage the symptoms and challenges associated with the disease.

The use of mobile health (mHealth) apps contributes to improving health literacy and facilitating communication between patients and their care providers [27]. Moreover, it improves patient well-being and helps caregivers make informed clinical decisions [28]. In fact, the use of such apps not only benefits patients while receiving treatment but also provides tools such as follow-up care and self-management for breast cancer survivors [29]. Patients’ need for self-management techniques is crucial, as it helps them to make their lives better by complying with the treatment needed and, as a result, accepting the disease [30].

However, despite the high number of downloads and star ratings of health apps, including those specifically targeting breast cancer, several challenges remain with respect to their quality and security. Evidence from the literature reports on the existence of poor-quality health apps that fall short with respect to (1) following evidence-based health guidelines and best practices, (2) involving experts and consumers in their development, and (3) demonstrating effectiveness based on empirical evidence, all of which ultimately can be potentially harmful to their users [14,31-33].

Additionally, health apps have been facing critical challenges related to their privacy, confidentiality, and security [14,34,35], especially given their nature of handling sensitive, personal, and health-related data [36]. These challenges have been magnified with the rise of cyberattacks through apps and mobile devices [37] and further highlighted by recent regulations such as the General Data Protection Regulation for member states of the European Union [38]. Such a regulation assesses the privacy score of mobile apps and identifies or measures apps’ privacy based on 14 components [39].

As the uptake of these apps increases, it becomes imperative for users to evaluate their quality and safety [40]. Despite the high prevalence of breast cancer among the population of the MENA region, evidence regarding the quality, privacy, and security of breast cancer apps available to Arabic speakers remains poor. This study aimed to conduct a systematic assessment of mobile breast cancer apps available for Arabic speakers to evaluate their functionality, quality, security, and data safety. To the best of our knowledge, no previous study has addressed this gap.

## Methods

### Overview

Using a similar approach to several recent studies [41-45] and to ensure scientific rigor, this study conducted a systematic search and content analysis of mobile breast cancer apps available for Arabic speakers. We searched both Google Play and Apple App stores between December 18 and 24, 2022.

### Search Strategy

Initially, we used the Arabic search terms highlighted in Table 1 to search Google Play and Apple App stores. These search terms were selected based on earlier work that was published concerning the top searched breast cancer topics by Arabic speakers using Google’s search engine [8]. To ensure rigor, the researchers searched the app stores both through (1) the web interface and (2) natively on devices running the relevant operating system, thus mimicking how end users will discover such apps.

**Table 1:** The terms used to search for breast cancer apps available to Arabic speakers and their English translations.

Arabic term	Translated term
سرطان الثدي	Breast cancer
فحص سرطان الثدي	Breast cancer screening
علاج سرطان الثدي	Breast cancer treatment or therapy
كشف سرطان الثدي	Breast cancer detection or screening
مرض سرطان الثدي	Breast cancer disease
أعراض مرض سرطان الثدي	Symptoms of breast cancer
فحص الثدي	Breast screening

### Eligibility

App eligibility was determined by 2 independent researchers blinded to each other’s decisions, and the apps were initially screened based on the app’s name, the provided screenshots, and the app’s description. Discrepancies between researchers were resolved through consensus. Apps were included if they were free of charge, provided content and support for Arabic speakers, and were designed for use by consumers or patients; all apps were considered regardless of release or last update dates (Textbox 1). Apps were excluded if they were paid or were subscription-based, did not support Arabic speakers, or were designed for use by clinicians or health care workers.

**Inclusion and exclusion criteria for the apps.**

**Inclusion criteria**

- Free of charge
- Available on Google Play or Apple App store
- Designed for use by consumers or patients
- Support Arabic speakers
- Considered regardless of release or last update dates

**Exclusion criteria**

- Paid or subscription based
- Designed for use by clinicians or health care workers
- Does not support Arabic speakers

### Data Extraction and Evaluation

Initially, all information provided by the app developers in the app stores was extracted to evaluate the descriptive features and the general characteristics of the included apps, which included the platform, developer name, update date, ratings, number of reviews, number of downloads, app category, and app permissions, as reported by the app developers. Afterward, 2 independent researchers downloaded the apps on their smartphones to assess the quality and privacy risks of the included apps.

We evaluated the quality of the included apps using a standardized form, the Mobile App Rating Scale (MARS), focusing on the following 4 dimensions: engagement, functionality, aesthetics, and information quality [46,47]. All scores were compared among 2 researchers, and the average score for each dimension was reported. To evaluate the apps’ privacy risks, we assigned scores to the permissions requested by the apps as reported by the app developers. The scores were informed by previous research, where the score risk is 0 for nonthreatening, 0.5 for potentially threatening, and 1 for threatening permissions [48]. Such permissions include access to restricted data, such as system state and user contact information, and restricted actions, such as connecting to a paired device and recording audio [49]. The 2 researchers independently carried out this evaluation and were unaware of each other’s scores; any discrepancies were resolved through consensus.

## Results

### Overview

**Figure 1:**
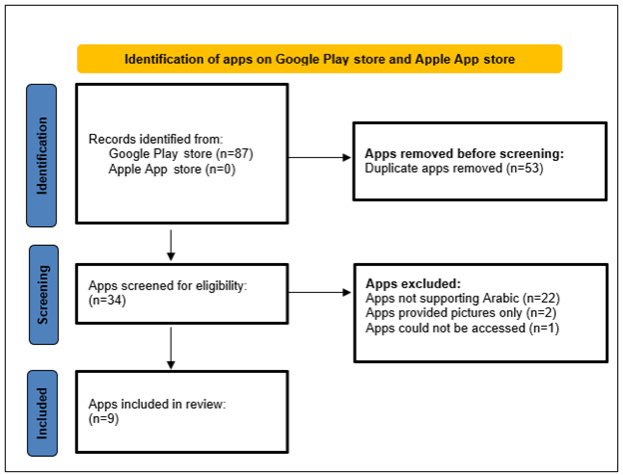
The flow diagram of the systematic search process to identify the relevant apps.

The researchers followed the systemic steps, highlighted in Figure 1, resulting in the inclusion of 9 apps, all of which are Android apps found on the Google Play store.

Overall, the included apps were indicated to be appropriate for all ages and were either in the medical, education, lifestyle, personalization, or health and fitness categories as per Google Play store categorization (Table 2). Our investigation suggests that the apps were all informational in nature, mainly providing information about breast cancer. None of the included apps had a language option to make it available in more than 1 language.

At the time of data collection, the results show that the included apps had low overall downloads (>10 to >1000) and more than half (6/9, 67%) were supported by advertisements. Only 5 apps had reviews, with an average of 10.60 reviews and an average star rating of 4.78. Only 1 app was last updated in 2019, while the remaining apps were updated in the past 2 years.

**Table 2:** Characteristics of the included breast cancer apps in the Google Play store.

App number^a^	Stars	Reviews, n	Advertisement supported	Downloads, n	Rating^b,c^	Updated on^d^	Category^b^
1	5	10	No	>100	3+	May 11, 2021	Medical
2	4.9	23	Yes	>1000	3+	July 26, 2019	Medical
3	—^e^	—	Yes	>100	3+	April 18, 2020	Health and fitness
4	4.2	6	Yes	>1000	3+	November 3, 2020	Education
5	5	8	No	>500	3+	May 11, 2021	Medical
6	4.8	6	No	50	3+	July 11, 2022	Medical
7	—	—	Yes	500	3+	August 9, 2021	Lifestyle
8	—	—	Yes	10	3+	October 8, 2022	Personalization
9	—	—	Yes	10	3+	October 8, 2022	Personalization

^a^Arbitrary number to mask app name.

^b^Based on Google Play store.

^c^Content of apps considered suitable for age group indicated per Google Play store rating.

^d^At data collection.

^e^Not available.

### App Permissions and Controls

As described in the Google Play store, the majority of the included apps (8/9, 89%) were requesting dangerous access permissions, including access to storage, media files, and camera permissions (Table 3). Additional permissions were exposed after the researchers downloaded the apps on the testing devices. These permissions included receiving data from the internet; viewing network connections; having full network access; running at start-up; controlling flashlight and vibration; preventing the device from sleeping; reading badge notifications; running foreground services; advertising permissions; reading location from media; playing and installing referrer application programming interface; and lastly, pairing with Bluetooth devices (Table S1 in Multimedia Appendix 1). The researchers considered all permissions and added them up to calculate the final risk score (Table 4).

On average, the security score for the included apps is 3.22 (total points possible: 9.5). The security scores ranged between 0 and 7.5; only 2 apps had a total security score of 0. The apps requested potentially dangerous permissions, namely full network access, advertising ID permission, read location from media collection, precise location (GPS and network-based), take pictures and videos, read the contents of the USB storage, modify or delete the contents of the USB storage, and view Wi-Fi connections.

**Table 3:** Included apps’ permissions as described in the Google Play store.

App number	Location			Camera		Photos and media			Storage			Wi-Fi connection
	Approximate location (network based)	Precise location (GPS and network based)	Take pictures and videos		Read the contents of your USB storage		Modify or delete the contents of your USB storage	Read the contents of your USB storage		Modify or delete the contents of your USB storage	View Wi-Fi connections	
1												
2											✓	
3	✓	✓	✓		✓		✓	✓		✓	✓	
4												
5												
6			✓					✓			✓	
7								✓			✓	
8												
9												

**Table 4:** The security score per app based on its permissions requested or declared.

Permission	Weight^a^	App								
		1	2	3	4	5	6	7	8	9
Receive data from the internet	0.5	0	0	1	1	0	1	1	1	1
View network connections	0.5	0	1	1	1	0	1	1	1	1
Full network access	1	0	1	1	1	0	0	1	1	1
Run at start-up	0	0	0	1	1	0	1	1	1	1
Control flashlight	0	0	0	1	0	0	0	0	0	0
Control vibration	0	0	0	1	1	0	1	1	1	1
Prevent device from sleeping	0	1	0	1	1	0	1	1	1	1
Read badge notification	0	0	0	0	1	0	1	1	1	1
Run foreground service	0	0	0	0	1	0	0	1	1	1
Advertising ID permission	1	0	0	0	0	0	1	0	1	1
Read location from media collection	1	0	0	0	0	0	1	1	0	0
Play install referrer API^b^	0	0	0	0	0	0	1	1	0	0
Pair with Bluetooth devices	0	0	0	0	0	0	1	1	0	0
Approximate location (network-based)	0.5	0	0	1	0	0	0	0	0	0
Precise location (GPS and network-based)	1	0	0	1	0	0	0	0	0	0
Take pictures and videos	1	0	0	1	0	0	1	0	0	0
Modify or delete the contents of your USB storage	1	0	0	1	0	0	0	0	0	0
Read the contents of your USB storage	1	0	0	1	0	0	1	1	0	0
Modify or delete the contents of your USB storage	1	0	0	1	0	0	0	0	0	0
View Wi-Fi connections	1	0	1	1	0	0	1	1	0	0
Total security score per app	9.5	0	2.5	7.5	2	0	6	5	3	3

^a^The threat weight was calculated following the guidance provided by Olmstead and Atkinson [48].

^b^API: application programming interface.

### Data Safety

Only 3 apps provided information about data safety and how the data are handled (Table S2 in Multimedia Appendix 1). Specifically, only 2 apps reported not sharing user data with other companies or organizations; the same apps reported that their apps do not collect user data. On the other hand, only 1 reported sharing information such as location, health and fitness messages, as well as photos and videos. The 3 apps that declared information about data safety reported that the data are encrypted in transit. Only 1 app reported that the users can request to delete the data.

### Apps’ Quality Rating

The researchers used MARS to evaluate the included apps’ quality. MARS uses 4 dimensions to assess the apps: engagement, functionality, aesthetics, and information. The average overall score rating of the included apps was 3.27 (Table 5). Notably, the evaluation showed that all the apps had a high rate in the functionality dimension, where they all scored 4.75; as for the engagement dimension, all apps had a meager score with an average of 2.32. Considering the other 2 dimensions, aesthetics and information, we can see that the scores vary between apps, where some have a high score and others have a low score, with an average of 3.00 and 3.04, respectively.

**Table 5:** Mobile App Rating Scale evaluation for Arabic breast cancer apps.

App number	Engagement	Functionality	Aesthetics	Information	Overall
1	2.30	4.75	3.67	3.87	3.60
2	2.40	4.75	3.00	3.00	3.30
3	2.40	4.75	3.00	2.25	3.10
4	2.40	4.75	3.33	2.50	3.20
5	2.40	4.75	3.33	3.50	3.50
6	2.40	4.75	3.67	4.00	3.70
7	2.20	4.75	3.00	2.75	3.20
8	2.20	4.75	2.00	2.75	2.90
9	2.20	4.75	2.00	2.75	2.90
Total mean score	2.32	4.75	3.00	3.04	3.27

## Discussion

### Principal Findings

To the best of our knowledge, this work summarizes the most extensive collection of the currently available free-of-charge breast cancer apps for Arabic speakers on the Google Play and Apple App stores. Interestingly, this systematic investigation reveals that, at the time of data collection, no breast cancer apps were identified as available to Arabic speakers in the Apple App store. Additionally, none of the apps at the time of data collection provided language options, which can hinder the possibility of translating to multiple languages. Overall, the results of this research showed a lack of breast cancer apps available to Arabic speakers compared to apps available in Turkish [50] and Korean [51] for example.

The analyzed apps in this study are available for free, with the majority of these apps (6/9, 67%) being supported by advertisements. The apps varied in what they are able to access and control on the devices, as shown in Table 3. A total of 44% (4/9) apps use Wi-Fi, 33% (3/9) can access the device’s storage, 22% (2/9) have access to the device’s camera, and 11% (1/9) are able to access the media as well as the location. Only 33% (3/9) of the apps stated how the collected data would be used (Table S2 in Multimedia Appendix 1), while the rest of the apps did not specify any information.

The results of this study demonstrate that the quality of the considered apps is highly “functional” but less “engaging” (Table 5). The average overall score rating of the included apps (3.27) is slightly above average, suggesting that the majority of the apps may not be considered exceptional by consumers. While there seem to be positive aspects to the included apps, there is still room for improvement. Considering Arabic content related to breast cancer, recent evidence suggests the low quality of informational videos available on YouTube despite the high number of views [9]. Our findings provide evidence suggesting that nearly average-quality information content about breast cancer is available to Arabic speakers.

A recent Spanish study tested 6 apps (2 on iOS, 5 on Android, and 1 on both) using the MARS framework. The results of the study showed relatively diverse scores, with an objective quality mean score of 3.06 and a subjective quality mean score of 1.96 [52]. Another study evaluated the quality of mHealth apps for educational purposes in Iran using the MARS framework. The study reported a mean score of 4.01 for quality and 3.08 for subjective quality [53]. Turkish apps were also evaluated using the MARS framework, and the study found an average score of 3.31 [50], which is similar to the Spanish study’s results.

Although many research papers have addressed the importance of using mHealth apps to improve patients’ health, provide educational materials, enhance communication between patients and caregivers, and achieve a successful recovery, these studies have stated that such apps are exposed to several challenges and threats as well. For instance, middle- and high-income households had more access to the internet on their mobile devices compared to those with low incomes [28]. Therefore, patients and caregivers who have no or limited access to the internet may not be able to use the app’s features effectively, or they might not prefer to use a mobile phone for health-related purposes in general.

Another concern is that the process of storing and transferring personal health data through a mobile app could be insecure and might cause serious security and privacy issues [28,54]. Thus, mobile apps should be designed to accommodate a wide range of possible users while considering health knowledge, different levels of cultural needs, and linguistic requirements. Additionally, recent research suggests that assessing the apps’ quality, safety, and usability by involving patients and health care professionals will result in an ideal app that meets patients’ requirements and enhances the app’s overall safety as well [20].

The literature on breast cancer smartphone apps suggests that these apps can be a valuable resource of information for individuals with the disease [55]. These apps provide information on treatment options, support groups, and local resources. The apps also allow individuals to track their symptoms, set reminders for appointments, and record their progress throughout treatment [56]. Such features can significantly contribute to enhancing patients’ well-being [57]. Additionally, studies have found that these apps can improve communication with health care providers and improve self-efficacy and quality of life [30,58]. However, it is important to note that the quality and accuracy of the information provided by these apps can vary, and it is recommended to consult with a health care professional before using any app for managing a medical condition.

A recent study on breast health and breast cancer apps notes that although apps appear to be competitive and useful for patients, some major features have to be considered while developing these apps [59]. The features include notifications, reminders, symptoms tracking, and recording. The study also suggests designing the apps to be user-friendly, even for low-literacy patients, by adding an audio feature (text-to-speech). Developing features with audio support will not only help patients with low literacy but can also support multiple languages.

### Recommendations and Implications for Practice

#### Privacy and Security

We would recommend that the developers of the apps be more transparent and state how the data will be used and that they should not have access to unnecessary data. We recommend that future breast cancer apps be available to Arabic speakers to justify the need for the permissions requested while also transparently disclosing the data safety handling measures to the app users. Security and privacy of apps are considered major requirements as they are accountable for sensitive patient data such as prescriptions, treatments, etc. Thus, to come up with robust apps that could ensure privacy and security appropriately, more evaluation techniques, as well as security mechanisms, should be analyzed and implemented on Arabic apps, in particular, to assess, measure, and control the apps’ security and privacy [60].

#### Quality and Engagement

Involving patients and health professionals in the app design phase is crucial. Several studies have addressed the idea that health applications should be developed and designed based on the combined efforts of health professionals, related academics, and patients [61]. To raise the quality of breast cancer apps, the inclusion of utility features such as appointment booking for mammograms and web-based consultations becomes necessary. In addition, it is recommended to improve health apps’ engagement by focusing on specific components such as personalized content, data visualization, reminders and notifications, educational material, self-management functions, and goal-setting features [62]. Providing users and patients with proper communication features and a well-designed interface leads to an ideal user experience as a result [30].

### Study Strengths and Limitations

Similar to other studies, a rigorous multistep methodology mimicking systematic reviews is used in this study to assess the breast cancer apps that are available to Arabic speakers. Apps were thoroughly searched through both the web interface as well as the app stores natively on the devices, mimicking how end users will discover such apps. While the results provide an indication of the quality of the evaluated apps, additional investigations are required to consider patients’ perspectives about their views about the quality as well as the utility of such apps. Future studies can also involve rigorous assessments with respect to the security measures applied by breast cancer apps available to Arabic speakers.

This study only considered the publicly available apps and may have missed apps that are “prescribed” to patients or consumers or those that are developed locally by health care organizations. Another limitation, which is inherent to the search strategy used in this work as well as similar other work [63], is the fact that the search algorithms used by the app stores are nontransparent and can change without the public’s knowledge, potentially undermining the reproducibility of the outcomes. Lastly, the current state of the results as revealed by this work is likely to change quite rapidly since apps are regularly released, updated, and retired.

### Conclusions

The battle against breast cancer is not over yet, and breast cancer apps can serve as valuable resources in this ongoing fight. The results of this systematic and thorough examination of breast cancer apps available for Arabic speakers reveal their limited existence at the time of study. The investigations evaluated these apps through the lenses of quality, privacy, and security, revealing that the included apps are rated as highly “functional” but at the same time are less “engaging.” The investigations also reveal that some apps were accessing unnecessary data and collecting information that was not relevant to the purpose of the app.

Developers of breast cancer apps that cater to Arabic speakers must focus on consumers’ preferences, demographics, usability, and the interface of their apps, as well as enhance measures and mechanisms of privacy and security for their apps. The low number of breast cancer apps available to Arabic speakers, as revealed in this study, should be a call to action for many health care organizations and developers to collaboratively develop information-rich, usable, functional, engaging, and secure apps.

## Appendix

Multimedia Appendix 1 Included apps' declared permission and safety declarations.

## Abbreviations

MARS: Mobile App Rating Scale
MENA: Middle East and North Africa
mHealth: mobile health

## Data Availability

All data generated or analyzed during this study are included in this paper and its supplementary information files.
