# Supplementary material for: Assessing the Quality, Privacy, and Security of Breast Cancer Apps for Arabic Speakers: Systematic Search and Review of Smartphone Apps
Source: JMIR Cancer. 2024 Jan 16;10:e48428. doi: 10.2196/48428 (PMC10828940; doi:10.2196/48428)
Supplement: Multimedia Appendix 1 [file cancer_v10i1e48428_app1.docx]

**Table S1.** Included apps’ declared permissions when downloaded on device.

| *App number* | *receive data from Internet* | *view network connections* | *full network access* | *run at startup* | *control flashlight* | *control vibration* | *prevent device from sleeping* | *read badge notification* | *run forground service* | *advertising ID permission* | *read location from media collection* | *play install referrer API* | *Pair with Bluetooth devices* |
| --- | --- | --- | --- | --- | --- | --- | --- | --- | --- | --- | --- | --- | --- |
| 1 | - | - | - | - | - | - | ✓ | - | - | - | - | - | - |
| 2 | - | ✓ | ✓ | - | - | - | - | - | - | - | - | - | - |
| 3 | ✓ | ✓ | ✓ | ✓ | ✓ | ✓ | ✓ | - | - | - | - | - | - |
| 4 | ✓ | ✓ | ✓ | ✓ | - | ✓ | ✓ | ✓ | ✓ | - | - | - | - |
| 5 | - | - | - | - | - | - | - | - | - | - | - | - | - |
| 6 | ✓ | ✓ | - | ✓ | - | ✓ | ✓ | ✓ | - | ✓ | ✓ | ✓ | ✓ |
| 7 | ✓ | ✓ | ✓ | ✓ | - | ✓ | ✓ | ✓ | ✓ | - | ✓ | ✓ | ✓ |
| 8 | ✓ | ✓ | ✓ | ✓ | - | ✓ | ✓ | ✓ | ✓ | ✓ | - | - | - |
| 9 | ✓ | ✓ | ✓ | ✓ | - | ✓ | ✓ | ✓ | ✓ | ✓ | - | - | - |

**Table S2.** Included apps’ safety declarations.

| **App number** | **Data sharing with 3rd Party** | **Data collection** | **Data encryption in transit** | **Data deletion requests** |
| --- | --- | --- | --- | --- |
| 1 | NA | NA | NA | NA |
| 2 | NA | NA | NA | NA |
| 3 | NA | NA | NA | NA |
| 4 | NA | NA | NA | NA |
| 5 | NA | NA | NA | NA |
| 6 | Location, Health and fitness messages, photos and videos, Audio, Files and docs, App activity, App info and performance, Device or other IDs | Location, Health and fitness messages, photos and videos, Audio, Files and docs, App activity, App info and performance, Device or other IDs | ✓ | ✓ |
| 7 | NA | NA | NA | NA |
| 8 | The developer says this app doesn’t share user data with other companies or organizations | The developer says this app doesn’t collect user data | ✓ | The developer doesn’t provide a way for you to request that your data be deleted |
| 9 | The developer says this app doesn't share user data with other companies or organizations | The developer says this app doesn’t collect user data | ✓ | The developer doesn’t provide a way for you to request that your data be deleted |
